# The vascular connector, design of a new device for sutureless vascular anastomosis

**DOI:** 10.1186/s13022-014-0008-4

**Published:** 2014-11-22

**Authors:** Lulzim Vokrri, Xhavit Krasniqi, Arsim Qavdarbasha, Nexhmi Hyseni, Philippe Cinquin, Paolo Porcu, Carmine Sessa

**Affiliations:** Department of Vascular Surgery, University Clinical Center of Kosovo, Medical Faculty of the University of Pristina, Boulevard “Dëshmoret e Kombit” nn; 10000, Pristina, Republic of Kosovo; Department of Vascular and Thoracic Surgery, University Hospital Centre of Grenoble; Faculté de Médecine, 38706 La Tronche Cedex, France

**Keywords:** Sutureless vascular anastomosis, New vascular connector, Vessel circuits

## Abstract

**Background:**

In recent years, several methods and new techniques have been studied and proposed for establishment of sutureless vascular anastomoses, streaming use of sutureless vascular surgery in the future.

**Presentation of the hypothesis:**

The new vascular connector (NVC) is a hypothetical design of a vascular device, proposed for creation and maintenance of sutureless vascular anastomosis. Implication of NVC would introduce a new device and technique in establishment of sutureless vascular anastomosis in which surgical approach is minimized and so post-operation disorders. It would eliminate need for suture; shorten clampage and operation time, consequently reducing stress for both, the surgeon and the patient. It enables the creation of vascular anastomosis fast, simple, safe, reliable, with satisfactory patency and stability of anastomosis.

**Testing the hypothesis:**

Efficacy of NVC needs to be evaluated in further studies, in order to be confirmed for clinical use. The effectiveness of NVC should be verified firstly in vitro and in vivo tests; and by animal experiments. The likelihood of its negative influence in thrombogenicity should be well evaluated.

**Implications of the hypothesis:**

Implication of the new vascular connector (NVC) would be of interest to both patients and the surgeon due to the following main achievements: 1) enables the creation of vascular anastomosis fast and simple, 2) significant shortening of clampage time of blood vessels and operation time-this assumption would be followed by reduced risk of operative and post-operative complications and length of hospital stay or admission to Intensive care unit, 3) safe and reliable, 4) compatible with any blood vessel and standard vascular graft, 5) using the NVC we will reduce in minimum need for replaced blood volume, 6) reduces the cost of treatment. It is anticipated that the NVC would provide shorter operation time and least operative and post-operative complications in creation of sutureless vascular anastomosis.

## Background

The first scientific attempt to restore vessel wall goes back to January 13, 1894, eight years before the description of the suture technique by Carrel. In the New York Medical Journal, R. Abbe has reported the possibility to replace an animal arterial conduit with hourglass shaped glass prosthesis [1]. Sutureless anastomotic devices are of more and more increasing interest in cardiovascular surgery. Since the beginning of cardiovascular surgery, proximal and distal anastomoses have been done with hand-held sutures based on the principles of the suture technique described by Alexis Carrel in 1902 [2]. In recent years, several methods and new techniques have been studied and proposed for establishment of sutureless vascular anastomoses, streaming use of sutureless vascular surgery in the future [3-10]. For fast and reliable recovery of patients with high risk surgery, especially for those with severe vascular disorders, new sutureless techniques should be feasible, fast, safe, and reliable with minimally invasive operations [11]. Special graft connectors and vascular device for connecting blood vessels and preserving integrity, continuity and patency of vascular lumen [12-17], have been applied for vascular anastomosis.

### General information of vascular anastomosis

**Figure 1:**
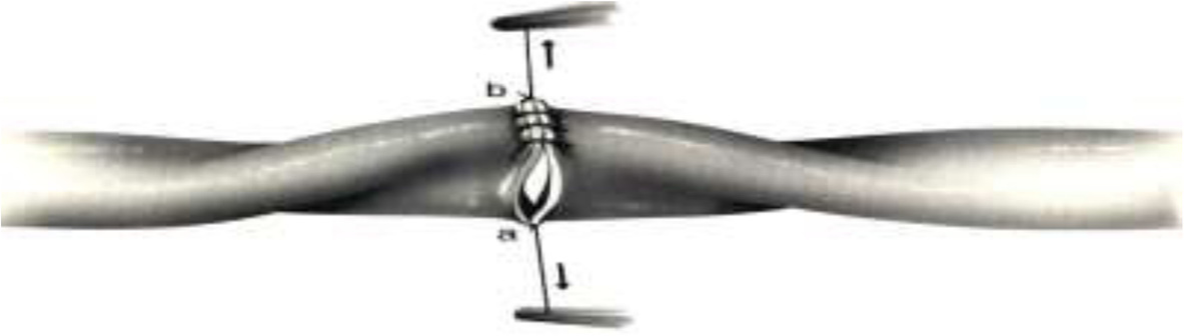
**Schematic representation of the** ***conventional vascular anastomosis.***

Vascular anastomosis is joining of two blood-carrying vessels. Some of the commonly practiced anastomoses are artery to artery, artery to vein, and artery to synthetic tube graft. Also, the two most common forms of an anastomosis are end to end; and end to side. In most cases vascular anastomoses are normally sutured conventionally - as the reference technique of sewing. We may even say that this technique is still considered as “golden standard” in cardiovascular surgery (Figure 1) [1]. In the pathologic arteries and limited access surgery or through minimal surgical approaches, the hand suture sometimes is difficult. This procedure depends on surgeon dexterity and requires time and great technical effort [3].

Therefore other efforts to sutureless vascular anastomosis of side-to-side, end-to-side, or end-to-end type, have been also tried with special devices, tools and equipment [3,4,12-18].

### Presentation of the hypothesis

As a new device for creation of a sutureless vascular anastomosis, is use of a new vascular connector. The hypothesis we propose here is that NVC may be an alternative treatment for sutureless vascular anastomosis. This hypothesis is based on the following points: 1) no need for suture, 2) shorter time of operation, 3) safety, 4) reliability, 5) patency and 6) stability of vascular anastomosis.

#### Proposal of a new vascular connector (NVC)

**Figure 2:**
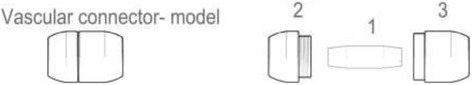
**Schematic representation of the** ***new vascular connector: 1. The inner tube with conical ends, 2. Male ring, and 3. Female ring.***

The NVC is composed of three parts, an inner tube with conical ends and the two outer rings, which serve to establish and maintain vascular anastomosis (Figure 2).

**Figure 3:**
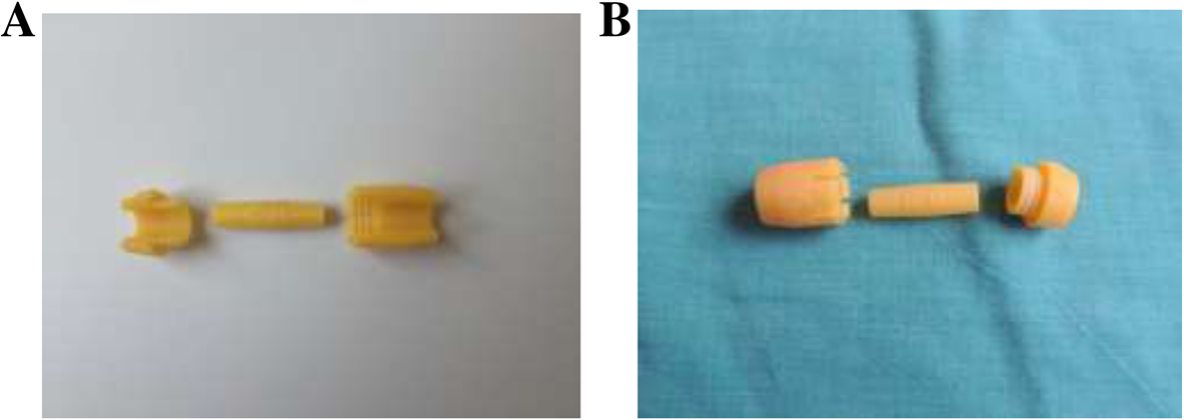
**View of the new vascular connector; A: External axonometric view of the NVC, B: External view of the NVC.**

NVC was made out of polymerized plastic (Photopolymer LS 600) and produced using a three dimensional (3-D) printer (Figure 3A and 3B). It is designed to make possible creation of end-to-end vascular anastomosis of various conduits: protheto-prosthesis, arterial-prosthetic, artery-artery, artery-vein and vein-vein. Vascular connector can be printed (produced) as required, for each type of blood vessel requiring vascular reconstruction. We have constructed a mathematical model for all dimensions; and have printed vascular connectors of various sizes of 0.1 mm increments from 4-7.5 mm.

The inner tube has a length of 20 mm. The maximal thickness of the wall of the inner tube in its centre is 0.3 mm, and in the ends is 0.1 mm. The central 3 mm of the inner tube is cylindrical in shape, and the rest is conical with a length of 8.5 mm, on both sides.

The central 3 mm cylindrical part of the internal tube was the maximum external diameter of the inner tube. The adjacent 8.5 mm on either side of this, are conical in shape with a gradual reduction in diameter. At the most distal end, there is a reduction of 0.4 mm in the diameter of the inner tube. It is precisely this change of diameter, which enables easy connection of blood vessels. Therefore, the external diameter of the inner tube is proportional to the internal diameter of the blood vessel or synthetic vascular graft. This allows end-to-end vascular anastomosis to be accomplished almost exactly around the line of artery-artery contact.

The size and the outer shape of the inner tube are directly proportional to the size and inner shape of the external rings. The length of the external rings is: masculine - 10 mm, feminine -15 mm. When connected they have a total length of 20 mm, equal to the length of the inner tube. The two outer rings of the vascular connector have three ratchet type teeth, which when they clasp between themselves with an axial slip, secure a leak proof connection of the anastomosis.

Masculine and feminine parts of the external conical ring are connected with the axial push, without rotation.

Safest and most consistent way to create a connection and leak proof sutureless anastomosis, in this technique, is to close fit the ends of the blood vessel over conical surface of inner tube, always maintaining the integrity of the vessel.

Regarding external compression of the vessel wall, the tested NVC was designed, calculated and printed for the vessel wall thickness of 0.5 mm, with an external compression of the connector rings of 15%, (0.075 mm)/2 = 0.0375 mm, in order to not compromise blood flow to the vasa vasorum.

### Clinical implication

**Figure 4:**
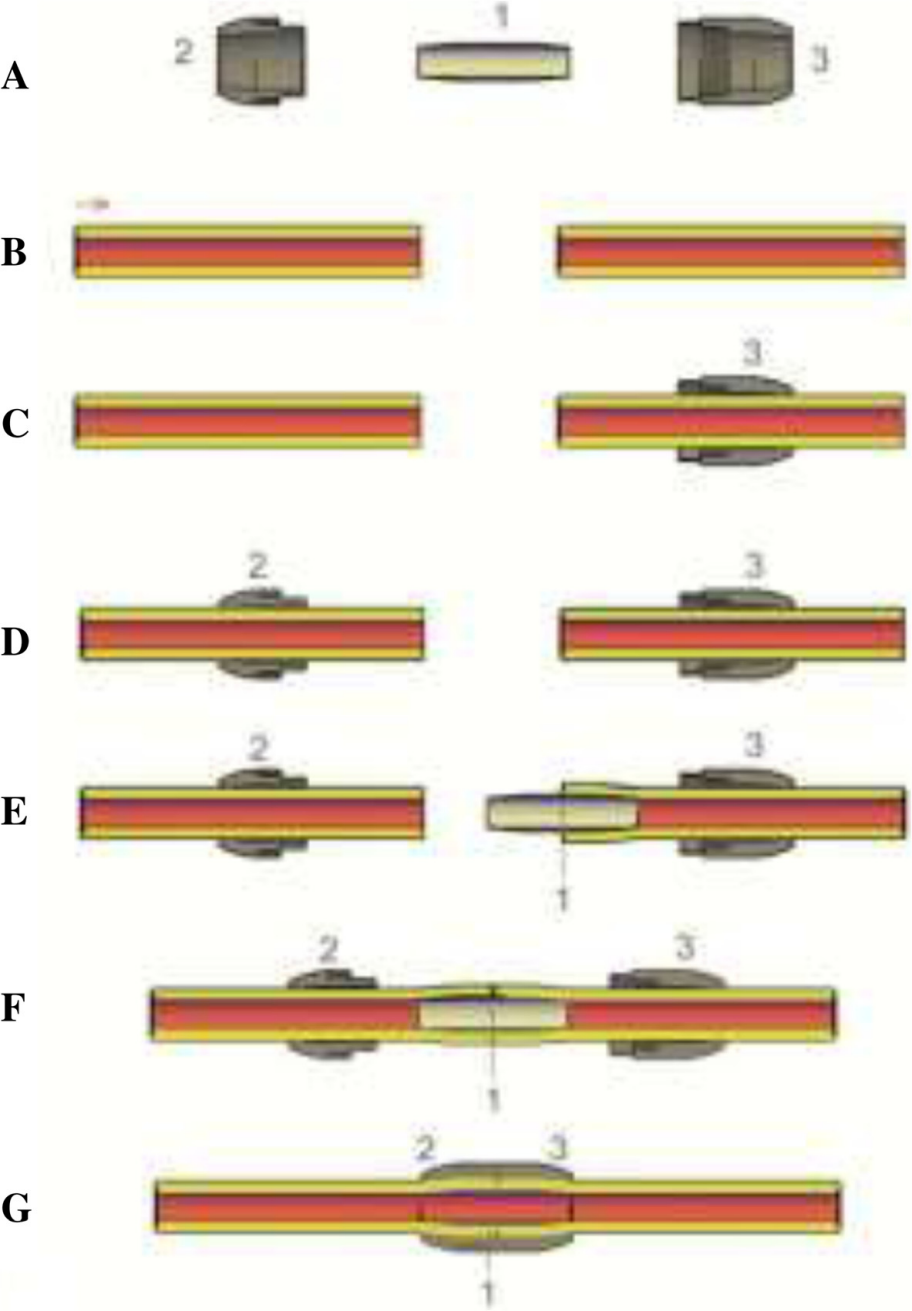
**Schematically presented stepwise procedure of sutureless vascular anastomosis by the NVC. A)** NVC in a front view (2D cut). **B)** Proximal and distal part of artery. **C)** *Female ring is located initially in the distal part of blood vessel.* ***D)*** *Male and female rings are situated in the proximal and distal part of blood vessel.* ***E)*** *Introduction of inner tube in the distal blood vessel.* ***F)*** *Introduction of inner tube in the distal and proximal blood vessel.* ***G)*** The final form of anastomosis.

NVC is proposed to create a sutureless vascular anastomosis. It bears less stress for both patient and surgeon. Details of the implementation of NVC to establish and maintenance the sutureless vascular anastomosis are schematically presented stepwise in Figure 4. In this technique, first part of the connector, distal ring, is located initially in the distal part of blood vessel (Figure 4C). Then, the second ring is situated in the proximal part of blood vessel (Figure 4D), to proceed with the introduction of fine inner tube with conical ends in the distal and proximal blood vessel (Figure 4E and F). The anastomosis is finalized with closure of outside positioned rings and interlocks both ends of the blood vessel or vascular graft prosthesis, without any gaps, to prevent leakage and eventual formation of late pseudo aneurysm. Consequently, two ends of the blood vessel are connected with each other without any suture (Figure 4G); Antiagregant drugs should be administered to minimize the risk of thrombosis in the postoperative period.

The following points support the NVC’s applicability for clinical use: 1) enables the creation of vascular anastomosis fast and simple. The NVC replaces the suture, as the most technically challenging part for vascular surgeons during the anastomosis procedures. Establishment of vascular anastomosis will take shorter time than existing techniques. 2) significant shortening of clampage time of blood vessels - this assumption would be followed by reduced risk of operative and post-operative complications and length of hospital stay or admission to Intensive care unit, 3) safe and reliable - creates permanent anastomosis with better blood flow, without migration and displacement of connector, without stenosis or pseudo aneurysm in anastomotic site, 4) using the new vascular connector we will reduce in minimum need for replaced blood volume, 5) compatible with any blood vessel and standard vascular grafts, 6) reduces the cost of treatment.

Comparison between conventional vascular anastomosis with sutureless anastomosis procedures is presented in Table 1.

**Table 1:** **Comparison between “conventional” and “sutureless vascular anastomosis”**

**Criteria**	**Conventional**	**Sutureless**
Operation duration	Longer	Shorter
Blood less during operation	More probable	Less probable
Safe and reliable	Less probable	More probable
Resistance of anastomosis (mechanical and pressure testing)	Less probable	More probable
Operative and post-operative complications	More probable	Less probable
Stress of surgeon and patient	More	Less
Reduce the cost of treatment	More	Less

## Testing the hypothesis

The NVC can be considered as a way to restore and to maintain vessel continuity; and it can be used either with biological or prosthetic grafts. Considering the application of this technique, further studies are needed to confirm its effect in clinical cases. The effectiveness of NVC should be verified firstly in in vitro tests, by animal experiments, and tests in cadavers. The likelihood of its negative influence on thrombogenicity should be well evaluated. When these concerns are cleared, we believe that NVC could be used as a new device for creation and maintenance of sutureless vascular anastomosis.

In order to test our hypothesis, we have performed ex vivo experiment on four freshly dissected carotid arteries of sheep. The arterial lumen diameter was measured using an electronic calliper. Four anastomosis were performed with the new vascular connector (n = 4;) and the other four (n = 4), using 6/0 prolene sutures and 13 mm needle. The carotid arteries were anastomosed end-to-end using the NVC 4.5 to 4.8 mm in diameter. This allowed end-to-end anastomosis to be accomplished almost exactly around the line of artery-artery contact. The time of completion of anastomoses was recorded. The end of the carotid arteries were ligated and clamped. This closed system was filled with 0.9% saline and the leakage of anastomosis was assessed. In addition, to complete this ex vivo experiment, a mechanical stress was applied at the anastomotic site of sutureless vascular anastomoses. The tension of detachment of anastomosis was measured by using a mechanical dynamometer. All parameters have been measured and recorded at the control group with sutured anastomoses.

## Test results

**Figure 5:**
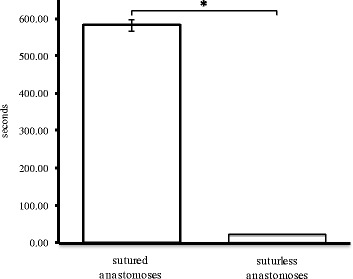
Anastomosis time (n=8, *Sig. at 5%; p =3,06 ×10 ^**-8**^ **).**

Ex vivo testing of the NVC was successfully completed. In all four cases, the sutureless anastomosis was completed in less than one minute. The mean duration of sutureless anastomosis was 23 seconds, whereas for manually sutured anastomoses it was 583.2 seconds. Compared to manually sutured anastomosis, duration of sutureless anastomosis was significantly shorter (p < 0.05) (Figure 5).

**Figure 6:**
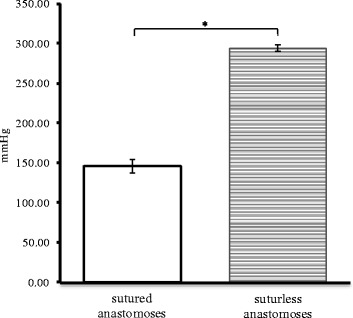
***Maximum pressure resistance in the anastomotic*** site (n=8, * Sig. at 5%; p = 6,89× 10 ^**-8**^ **).**

Maximum pressure resistance at the anastomotic site - No leakage occurred at the sutureless anastomotic site on internal pressures of up to 295 mmHg, versus manually sutured anastomoses where the leakage occurred at 146.25 mmHg, (p < 0.05). The sutureless anastomoses were significantly higher in internal pressure resistance, then the sutured anastomoses. Both tests were performed in the eight samples immediately after the surgery (Figure 6).

**Figure 7:**
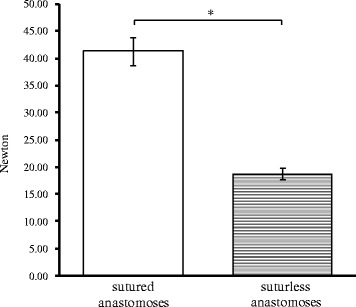
Detachment force of anastomosis (n=8, * Sig. at 5%; p =2,83 ×10 ^**-6**^ **).**

Detachment test of force of anastomosis-Immediately after the completions of the anastomosis, the mean forces of tension resistance were 18.75 N for the sutureless anastomoses and 41.25 N for manually sutured anastomoses. According to these results, when compared to sutureless anastomoses, manually sutured anastomoses had a significantly higher tensile strength (p < 0.05), (Figure 7).

## Discussion

Several experimental studies, using diverse devices and mechanisms, for the creation of sutureless vascular anastomosis have been conducted; but unsatisfactory results have limited the wide application of such methods in human clinical practice [3-10,12-21].

The new vascular connector suggested in this work provides shorter time, safety, reliability, patency and stability of anastomosis.

Since new vascular connector is a very fine device, it ensures perfect match of both ends of the blood vessel, or vascular graft prosthesis, establishing effectively a sutureless vascular anastomosis. In addition, after the closing of the vascular connector rings at the outer part of the vessel, we will ensure safety of the anastomosis and prevention of possible migration of proximal and distal part of the blood vessel or vascular graft prosthesis and bringing practically to zero, the leakage of blood around created anastomosis. This technique can be used for blood vessels of small, medium and large diameter, but can also be modified for use in anastomosis of very small vessels, using super fine vascular connectors of different sizes.

Moreover, it may open new windows for minimally invasive microsurgery and laparoscopy.

This method is described for end-to-end anastomosis, while a large portion of vascular anastomosis is end-to-side. If this new vascular connecting device and technique provides good results in the first experimental studies, it opens perspective of studies of application of the same device in form of letter T with different angles, for end-to-side anastomosis.

Such studies should evaluate the feasibility and efficiency by selecting the animals, e.g. sheep’s blood vessel cut circumferentially, one anastomosis would be created by traditional method and another using the new vascular connector device. The anatomical, hysto-pathological, and long-term clinical results of this vascular anastomosis should then be evaluated with a special attention.

Our initial ex vivo testing of NVC anastomosis demonstrates that the NVC has some advantages over sutured anastomosis regarding simplicity, and shorter time of creation, as it is faster to execute without qualitative repercussions. In practical terms, it is expected to reduce the time of vascular clamping.

The assessment of the leakage of anastomosis with the NVC ex vivo was done by use of liquid saline, which does not have the same characteristics as the blood. The viscosity of the 0.9% saline solution used for testing the leakage is 1.005×10^3^at 20° Celsius (°C), while the blood is 4×10^3^ at 37°C and leakage occurs more easily with water and liquid saline compare to blood [22]. The results show a perfect seal with the 0.9% saline despite very significant pressure applied to all vascular grafts anastomoses in this ex vivo experiment.

Significant manual stretch forces applied immediately after the procedure did not compromise the anastomoses with the NVC. Tensile strength tests confirmed the adequate mechanical strength of the anastomoses, but the manually sutured anastomoses were significantly more resistant. There are few possible points for study criticism, including greater number of cases in ex vivo experiment, and tests of creation of anastomosis on atherosclerotic model vessels, but it is a first step to show the feasibility of our model study.

## Conclusion

The new vascular connector can be considered as a way to restore and to maintain vessel continuity; and it can be used either with biological or prosthetic grafts. We think that the presented device may ultimately permit quick vascular anastomoses. The NVC appears to have following benefits in comparison with other techniques:

- Enables the creation of vascular anastomosis faster and simple. The new vascular connector replaces the suture, as the most technically challenging part for vascular surgeons during the anastomosis procedures.
- Significant shortening of clampage time of blood vessels - this assumption would be followed by reduced risk of operative and post-operative complications and length of hospital stay or admission to Intensive care unit.
- Safe and reliable - creates permanent anastomosis with better blood flow, without migration and displacement of connector, without stenosis or pseudo aneurysm in anastomotic site.
- Compatibility with any blood vessel and standard vascular graft.
- NVC will reduce in minimum need for replaced blood volume.
- Reduces the cost of treatment.

We believe this technique can be a step ahead and opens a new window for faster, simpler and easier surgery with a lower cost of treatment. The sutureless vascular connector device, tested in first ex vivo experiment, was demonstrated to be effective and safe; but it needs to be carefully evaluated through series of preclinical experiments to ensure that it provides satisfactory results for clinical trials.

## Abbreviations

NVC: New vascular connector
